# Supplementary material for: Thermal Shift Assay for Small GTPase Stability Screening: Evaluation and Suitability
Source: Int J Mol Sci. 2022 Jun 26;23(13):7095. doi: 10.3390/ijms23137095 (PMC9266822; doi:10.3390/ijms23137095)
Supplement: Supplementary file 1 [file ijms-23-07095-s001.zip › ijms-1756044-supplementary.pdf]

International Journal of *Molecular Sciences*

MDPI

*Article*

# Thermal Shift Assay for Small GTPase Stability Screening: Evaluation and Suitability

**Kari Kopra [1,*], Salla Valtonen [1], Randa Mahran [1], Jonas N. Kapp [2], Nazia Hassan [1], William Gillette [3], Bryce Dennis [4], Lianbo Li [4], Kenneth D. Westover [4], Andreas Plückthun [2] and Harri Härmä [1]**

[1] Department of Chemistry, University of Turku, Henrikinkatu 2, 20500 Turku, Finland; sallamaria.valtonen@gmail.com (S.V.), randa.r.mahran@utu.fi (R.M.), nazia.n.hassan@utu.fi (N.H.), harri.harma@utu.fi (H.H.)
[2] Department of Biochemistry, University of Zurich, Winterthurerstrasse 190, 8057 Zurich, Switzerland; j.kapp@bioc.uzh.ch (J.N.K.), plueckthun@bioc.uzh.ch (A.P.)
[3] Leidos Biomedical Research, Inc., Frederick National Laboratory for Cancer Research, 8560 Progress Dr., Frederick, MD 21702, USA; gillettew@mail.nih.gov
[4] Departments of Biochemistry and Radiation Oncology, University of Texas Southwestern Medical Center at Dallas, 5323 Harry Hines Blvd, L4.270, Dallas, TX 75390, USA; brycedennis@gmail.com (B.D.), lianbo.li@utsouthwestern.edu (L.L.), kenneth.westover@utsouthwestern.edu (K.D.W.)
* Correspondence: kari.kopra@utu.fi; Tel.: +358 50 328 5641

## Supplementary Materials and Methods

*Materials and Instrumentation*

Heptadentate ITC-TEKES-Eu(III)-chelate and nonadentate europium-chelate-9d, {2,2',2'',2'''-{[4'-(4'''-isothiocyanatophenyl)-2,2',6',2''-terpyridine-6,6''-diyl]bis(methylene-nitrilo)}tetrakis(acetate)}europium(III), used for $Eu^{3+}$-streptavidin (SA), $Eu^{3+}$-GTP, and $Eu^{3+}$-probe peptide ($NH_2$-EYEEEEEVEEEVEEE) conjugations, and the soluble quencher molecule, MT2, were obtained from QRET Technologies (Turku, Finland). Labels were used according to the manufacturer's instructions. Purifications and concentration determinations for $Eu^{3+}$-chelate-conjugated small molecules and AFDye 647 TFP ester (Fluoroprobes, Scottsdale,USA) conjugated GTP (AF647-GTP) were performed based on label concentration monitored against their appropriate standards [1-3]. NAP-5 columns were purchased from GE Healthcare (Uppsala, Sweden), and used for $Eu^{3+}$-SA purification according to the manufacturer's instructions. SA was purchased from BioSpa (Milan, Italy). Black Framestar 96-well plates from 4titude (Surrey, U.K.) were used in all thermal denaturation assays. White Corning 384-well low volume assay plates were used in all other assays. Nonhydrolyzable guanosine-5'-triphosphate (GTP) analogs, guanosine-5'-[(β,γ)-imido]triphosphate (GMPPNP), guanosine-5'-(γ-thio)-triphosphate (GTPγS), and guanosine-5'-[(β,γ)-methyleno]triphosphate (GMPPCP) were purchased from Jena Bioscience (Jena, Germany). Other reagents, including analytical-grade solvents, buffer components, SYPRO Orange (5000x), ANS (8-anilinonaphthalene-1-sulfonic acid), GTP, (guanosine-5'-diphosphate) GDP, guanosine-5'-monophosphate (GMP), and 1,1,3,3,3′,3′-hexamethylindodicarbocyanine iodide (HIDC) were from Sigma-Aldrich (St. Louis, MO, USA).

Reverse-phase liquid chromatography Dionex ultimate 3000 LC system (Dionex Corporation, Sunnyvale, CA, USA) and Ascentis RP-amide C18 column (Sigma-Aldrich, Supelco Analytical) were used for $Eu^{3+}$-GTP and $Eu^{3+}$-probe purification [1,4]. All measurements were performed using a Spark 20M from Tecan Life Sciences (Männedorf, Switzerland). Time-resolved luminescence (TRL) measurements for $Eu^{3+}$-GTP and $Eu^{3+}$-probe were performed at 615 nm, using 340 nm excitation wavelength (800 μs delay and 400 μs decay), and TR-FRET assays with AF647-GTP at 665 nm using 340 nm excitation (50 μs delay and 200 μs decay). SYPRO Orange and ANS fluorescence was measured using 485/590 nm and 350/490 nm excitation and emission wavelengths, respectively. Thermal denaturation assays were performed with a PTC-100 Programmable Thermal Controller (MJ Research, Inc., Watertown, MA).

*Protein production and Purification*

Expression and purification of recombinant Gαi protein has been described previously, and it was a kind gift from Arto Pulliainen (University of Turku) [5]. Truncated KRAS V14I (residues 1-169) lacking the *C* terminal residues, and DARPins K13 and K19 (kind gift from Terence Rabbitts, Institute of Cancer Research) were expressed and purified as described previously [6,7]. DNA constructs for the expression, and purification of iMet-KRAS (G-Hs.KRAS4b(1-169)) (Addgene #159539) and Ac-KRAS (Hs.KRAS4b(1-169))(Addgene #159690) [8], a human son of sevenless SOS1 catalytic domain (SOS$^{cat}$, 564–1048) and K27 DARPin have been previously described [2].

Gateway Entry clones for other proteins (G-Hs.HRAS(1-189), G-Hs.NRAS(1-189), GG-Hs.KRAS4b (2-188), GG-Hs.KRAS4b (2-188) G12C, GG-Hs.KRAS4b (2-188) G13D, GG-Hs.KRAS4b (2-188) Q61L, GG-Hs.KRAS4b (2-188) Q61R, and RhoA (1-182) were generated by standard cloning methods and incorporate an upstream tobacco etch virus (TEV) protease cleavage site (ENLYFQG) followed by the appropriate protein coding sequences. Sequence-validated Entry clones were sub-cloned into pDest-566 (Addgene #11517), a Gateway Destination vector containing a His6 and maltose-binding protein tag, to produce the final *E. coli* expression clones [9]. Proteins were expressed using the Dynamite/16 °C induction protocol,[9] and purified as described previously [2]. As an exception, GG-Hs.KRAS4b (2-188) G13D was purified in the presence of 250 μM GDP from the point of TEV protease digestion onward.

Avi-KRAS was produced using the coding sequence of human KRAS4B (residues 1-188) and it was cloned in a pQIq plasmid providing a N-terminal MRGS-8xHis-TEV-Avi-tag. Biotinylated KRAS was expressed in AVB100 *E. coli* (Avidity) for 16 h at 18 °C in Terrific Broth (TB) containing 2% (*v*/*v*) EtOH. Expression of KRAS was induced with 0.2 mM IPTG while expression of BirA, integrated in the bacterial genome, was induced with 0.4% (*w*/*v*) *L*-arabinose. All steps of protein purification were performed at 4 °C. Bacterial pellets were resuspended in lysis buffer (50 mM Tris-HCl pH 8.0, 10% (*w*/*v*) glycerol, 300 mM NaCl, 20 mM imidazole; 10 mM $MgCl_2$, 0.1 mM GDP containing 25 ng/ml DNAse, 1 mg/ml lysozyme, 5 μg/ml leupeptin, 48 μg/ml Pefabloc SC, and 1 μg/ml pepstatin-A) and lysed via sonication. After centrifugation (20,000× *g* for 30 min) and filtration (0.2 μm), KRAS was purified using metal-ion affinity chromatography (IMAC). Subsequently, the IMAC eluate was dialyzed against 50 mM HEPES pH 7.4, 10% (*w*/*v*) glycerol, 2 mM TCEP, 25 mM NaCl, 20 mM imidazole; 10 mM $MgCl_2$ involving cleavage of the *N*-terminal tag by the tobacco etch virus (TEV) protease. TEV protease and uncleaved protein were removed by reverse IMAC. KRAS was further purified by cation exchange (Mono S 5/50 GL) and size exclusion chromatography (HiLoad 16/600 Superdex 75 pg) to provide a monomeric protein.

**KRAS Nucleotide Exchange Activity and Inhibition Monitoring**

Nucleotide exchange assays were performed either with non-modified RAS proteins utilizing the QRET nucleotide exchange assay principle or utilizing a TR-FRET readout and Avi-KRAS [1,3,10,11]. All assays were performed in an assay buffer, containing 20 mM HEPES (pH 7.5), 1 mM $MgCl_2$, 10 mM NaCl, 0.01% Triton-X 100, 0.005% γ-globulins. All QRET assays were performed in 15 μL final volume using KRAS WT or G12C mutant at 15–100 nM concentration. All QRET assays were performed using an endpoint protocol by monitoring the TRL-signal multiple times during 60 min incubation. In a nucleotide exchange reaction, 10 nM $Eu^{3+}$-GTP and 2.5 μM MT2 quencher were used in a SOS$^{cat}$ (5 or 10 nM) induced reaction. The QRET assay was used for titrations with nucleotides (GTP, GDP, GMP, GMPPNP, GMPPCP, GTPγS, and ATP), small molecule inhibitors (ARS-853, ARS-1620, BI-2852, BI-2853, BAY-293, AMG-510, and MRTX849), and protein inhibitors (DARPins K13, K19, and K27). In all assays, studied nucleotides or inhibitors were first incubated for 5 min (KRAS WT) or 20 min (KRAS G12C) in 9 μL volume. Thereafter, 3 μL of detection solution ($Eu^{3+}$-GTP and MT2) was added, and finally the reaction was

initiated by the addition of $SOS^{cat}$ at 3 μL. TR-FRET assays were performed in 10 μL final volume using real-time detection monitoring. The studied protein binders (K13, K19, and K27) were incubated for 10 min in the presence of Avi-KRAS (50 nM) at 6 μL volume. $SOS^{cat}$ (0–10 nM) was added at 2 μL volume and the reaction was initiated by the addition of 2 μL of the detection solution (50 nM AF647-GTP and 5 nM $Eu^{3+}$-SA). Association kinetics with or without $SOS^{cat}$ were monitored at 665 nm for 90 min.

**Data Analysis**

In all assays, the signal-to-background ratio (S/B) was calculated as $\mu_{max}/\mu_{min}$ and coefficient of variation (CV%) (σ/μ)*100. In all formulas μ is the mean value, and σ is the standard deviation (SD). Data were analyzed using Origin 8 software and the half-maximal inhibitory concentration ($IC_{50}$) and the half maximal effective concentration ($EC_{50}$) values were obtained using standard sigmoidal fitting functions. Data were analyzed using Origin 8 software (OriginLab, Northampton, MA).

**Supplementary figures**

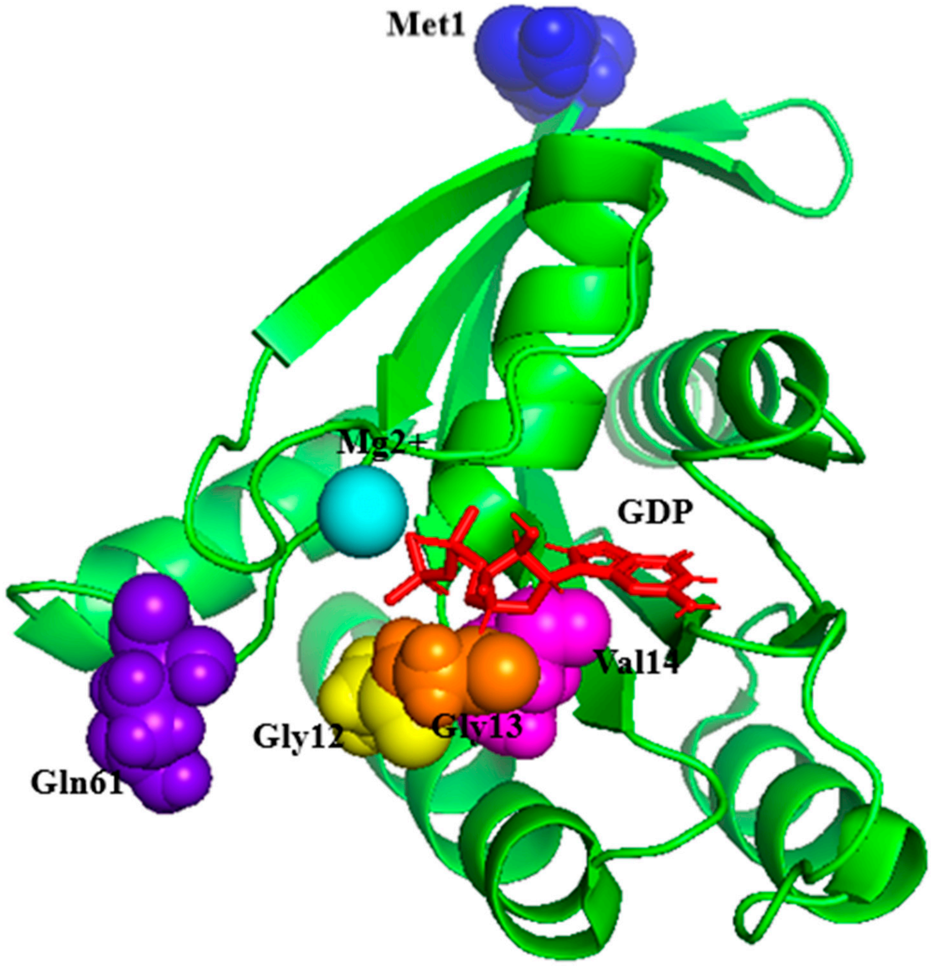


**Figure S1.** Crystal structure of GDP-bound human KRAS (PDB: 4OBE) marking the used mutations [12]. KRAS mutants used in this study are mainly located near $Mg^{2+}$ (cyan) and GDP (red) binding pocket. Gly12 (yellow), Gly13 (orange), Val 14 (magenta), and Gln61 (purple) all has effect on the known KRAS activities when mutated. Effect of *N*-terminal initiator methionine (Met 1, blue) was also studied.

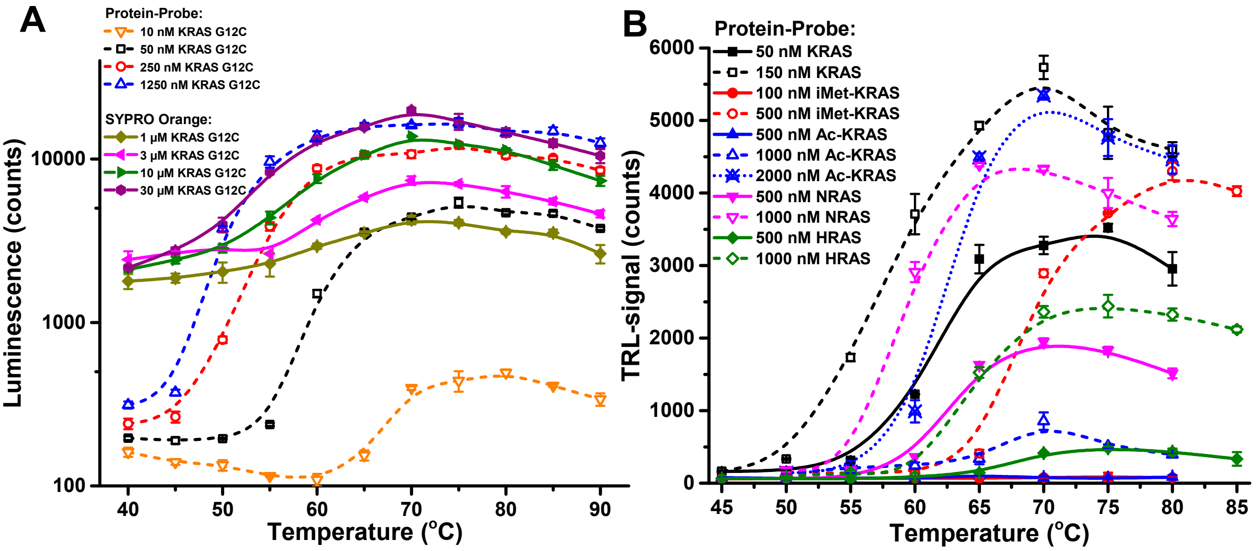


**Figure S2.** Thermal stability of various RAS forms in the Protein-Probe and SYPRO Orange assays. **A**) The detectability and signal levels of full length KRAS G12C in the Protein-Probe (10–1250 nM, dashed) and SYPRO Orange (1–30 μM, solid). In case of the Protein-Probe, 10 nM KRAS G12C was found as the lowest usable concentration giving S/B ratio over 3, and the S/B ratio saturated at about 250 nM KRAS G12C giving the S/B over 50. With SYPRO Orange, 1 μM KRAS G12C already produced a thermal curve, but the S/B of 3 was reached not before at 3 μM KRAS G12C. As the SYPRO Orange signal at RT was basically unaffected by the increase in KRAS G12C concentration, it increased the S/B ratio accordingly. B) The detectability of full length KRAS (0.05 or 0.15 μM, black), HRAS (0.5 or 1.0 μM, green), and NRAS WT (0.5 or 1.0 μM, magenta) and truncated Ac-KRAS (0.5, 1.0 or 2.0 μM, blue) and iMet-KRAS (0.1 or 0.5 μM, red) vary when assayed with the Protein-Probe. Full length KRAS WT (aa 2–188) was found the most detectable, as already 50 nM protein (solid) showed high TRL-signal, which was still increased at 150 nM (dashed). Truncated (aa 1–169) iMet-KRAS and Ac-KRAS were significantly less detectable. iMet-KRAS gave no TRL-signal increase at 100 nM (solid), but clear denaturation curve with 500 nM (dashed) concentration. Ac-KRAS was even less visible showing no or low TRL-signal at 500 nM (solid) or 1000 nM concentrations, but a clear thermal denaturation curve at 2000 nM (dotted). HRAS (aa 1–189) and NRAS (aa 1–189) were both moderately detectable at 500 nM (solid) and well detectable at 1000 nM concentration. Data represent mean ± SD (*n* = 3).

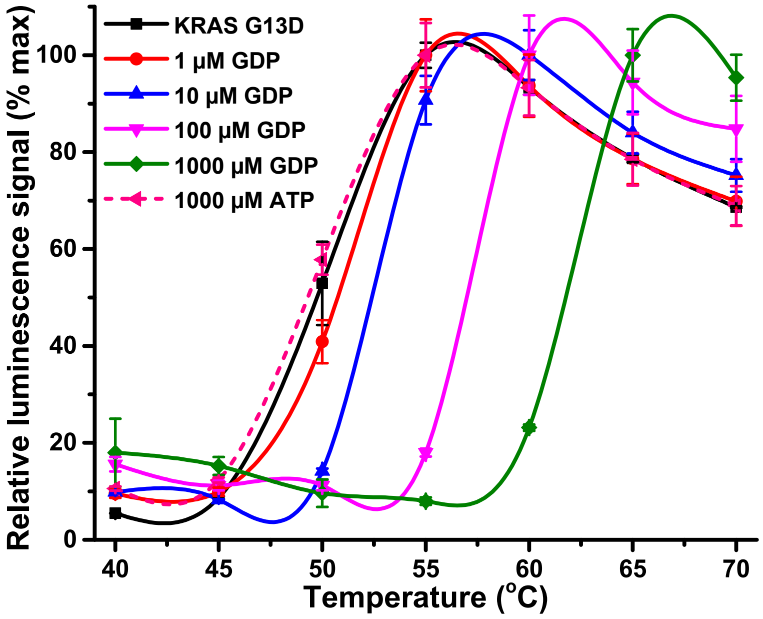


**Figure S3.** Thermal stability of KRAS G13D in the presence of GDP assayed with SYPRO Orange. KRAS G13D (3 μM) showed a GDP concentration-dependent thermal stability increase. 1 μM GDP (red) showed no KRAS G13D stabilization, while 10 (blue), 100 (magenta) and 1000 μM (green) GDP had a clear stabilizing effect over KRAS G13D alone (black), when assayed in the buffer with 1 mM $MgCl_2$. ATP (dashed pink) had no effect on thermal stability even at 1 mM concentration. Due to the relative high (3 μM) KRAS G13D concentration, no thermal stability saturation was reached even at the highest GDP concentration. Data represent mean ± SD (*n* = 3).

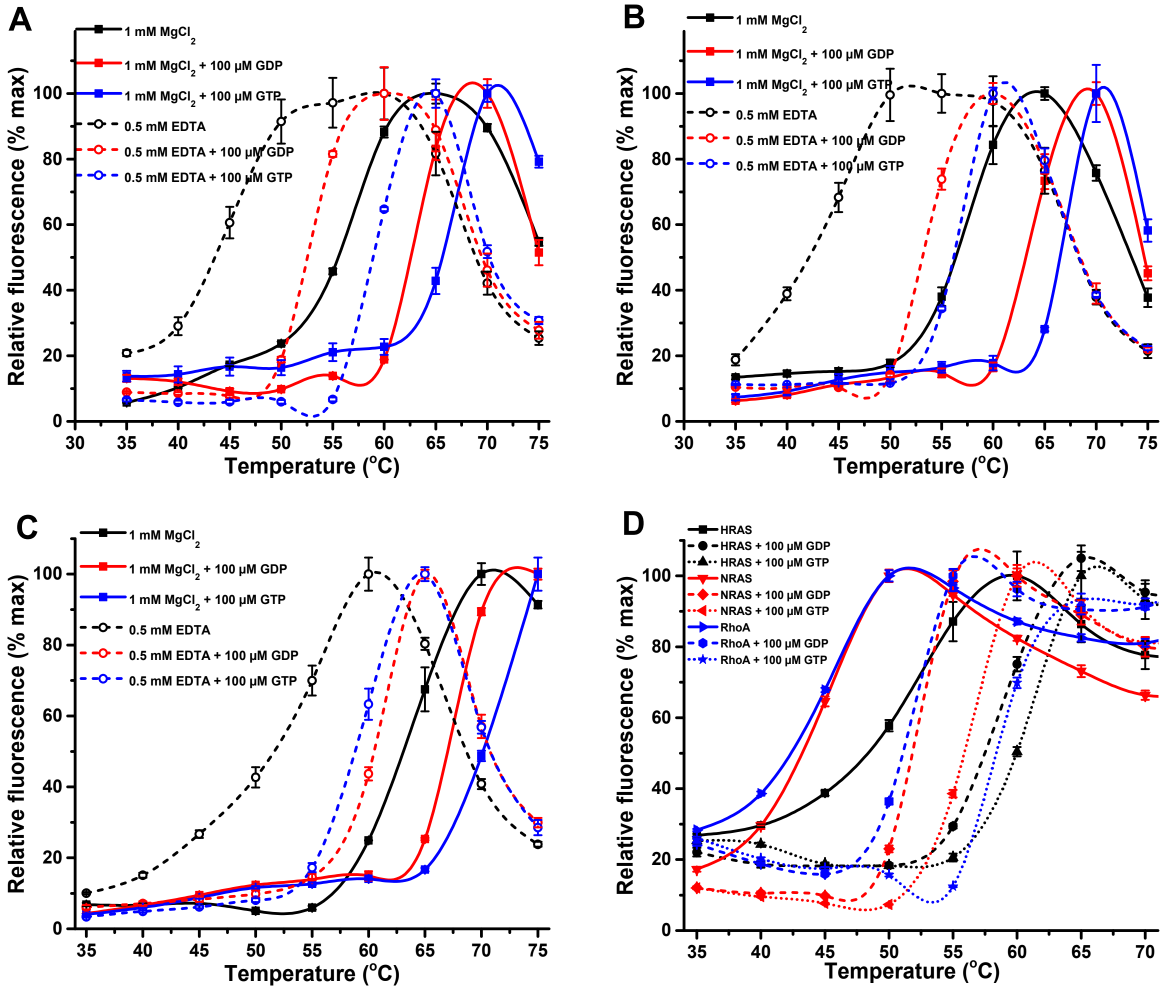


**Figure S4.** Thermal stability of RhoA WT, NRAS WT, and HRAS WT in the presence or absence of GDP/GTP. RhoA (**A**), NRAS (**B**), and HRAS (**C**) were assayed in a buffer with 1 mM $MgCl_2$ (solid) or 0.5 mM EDTA (dashed). SYPRO Orange assays with small GTPases (10 µM) were performed in the absence (black) or presence of GDP (red) or GTP (blue). RhoA (blue), NRAS (red), and HRAS (black) were also assayed in a buffer with 0.5 mM EDTA using ANS dye and without (solid) or with 100 µM GDP (dashed) or GTP (dotted) (**D**). The presence of 1 mM $MgCl_2$ stabilized all tested small GTPases in comparison to chelating conditions with 0.5 mM EDTA. RhoA, NRAS, and HRAS all showed a higher thermal stability increase in the presence of added GTP in comparison to GDP. The preference was most significant with RhoA in the absence of $Mg^{2+}$. These effects were dye independent, as similar effects were monitored both with SYPRO Orange and ANS dyes. Data represent mean ± SD (n=3).

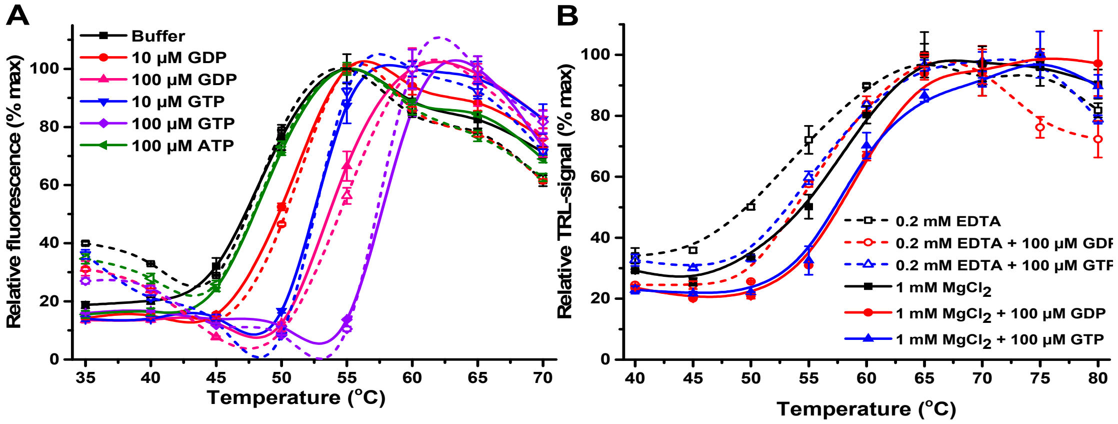


**Figure S5.** Thermal stability of KRAS V14I using SYPRO Orange and ANS, and G$\alpha$i stability using the Protein-Probe. **A**) KRAS V14I (10 μM) stability was assayed using SYPRO Orange (solid) and ANS (dashed) dyes, in the absence of nucleotide or in the presence of 10 or 100 μM GDP/GTP and 100 μM ATP. KRAS V14I was concentration dependently stabilized by GDP (red, magenta) and more significantly with GTP (blue, violet) in an assay buffer supplemented with 1 mM $MgCl_2$. ATP (green) had no effect on KRAS V14I stability. **B**) G$\alpha$i (50 nM) was studied in the presence of 1 mM $Mg^{2+}$ (solid) and in chelating conditions (0.2 mM EDTA, dashed). There were only a small destabilization of G$\alpha$i, caused by EDTA, and also only a small 100 μM GDP/GTP (red/blue) induced stabilization when the Protein-Probe assay was performed in either condition. Data represent mean ± SD (n=3).

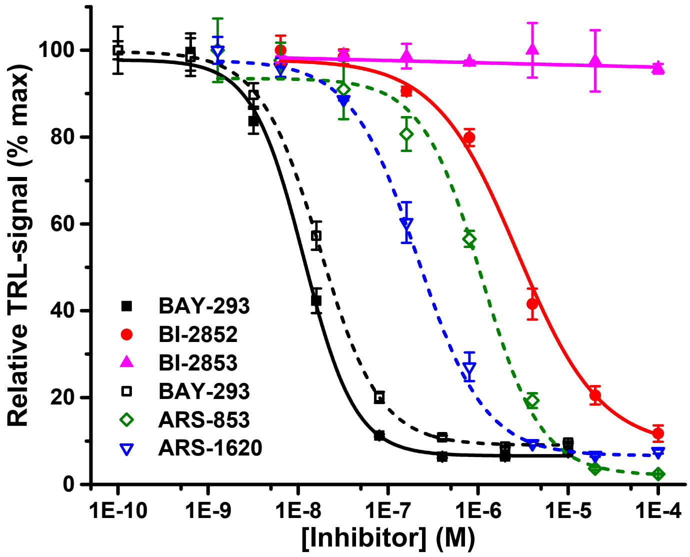


**Figure S6.** Nucleotide exchange inhibition assay with KRAS WT and G12C. Binding properties for a panel of inhibitors were determined using $SOS^{cat}$ (10 nM) induced nucleotide exchange assay with 100 nM KRAS WT (solid) and 100 nM G12C (dashed). BI-2852 (red, KRAS inhibitor), BI-2853 (magenta, non-specific), and BAY-293 (black, SOS inhibitor) were assayed. Similarly, KRAS G12C was assayed with ARS-853 (green, KRAS G12C inhibitor), ARS-1620 (blue, KRAS G12C inhibitor), and BAY-293 (black). BI-2852 showed KRAS WT inhibition ($IC_{50}$ value of 2.8 ± 0.7 μM) and $IC_{50}$ values for KRAS G12C with ARS-853 and ARS-1620 were 1.1 ± 0.1 and 0.21 ± 0.04 μM, respectively. BAY-293 showed KRAS-independent inhibition blocking the $SOS^{cat}$ function with an $IC_{50}$ values close to the $SOS^{cat}$ concentration of approximately 10 nM. The non-specific control compound, BI-2853, had no effect on KRAS nucleotide exchange activity. Data represent mean ± SD (n=3).

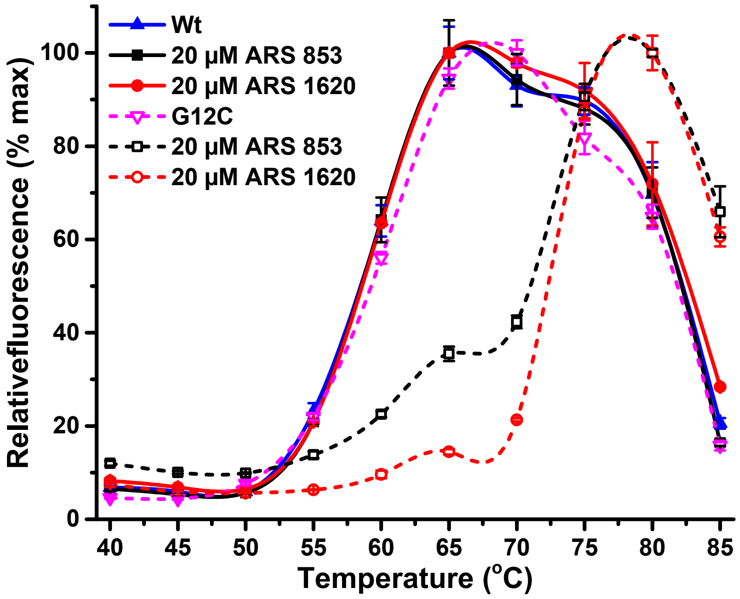


**Figure S7.** Thermal stability of KRAS WT and G12C with ARS-853 and ARS-1620 in the SYPRO Orange assay. Covalent inhibitors ARS-853 (black) and ARS-1620 (red) were assayed in 20 μM concentration with 10 μM KRAS WT (solid) or G12C (dashed). Neither compound altered the thermal stability of KRAS WT (compare solid lines). In comparison of 10 μM KRAS G12C (magenta) alone, ARS-853 and ARS-1620 both provided double transitions with a maximal thermal stabilization of approximate 13°C. Data represent mean ± SD (n=3).

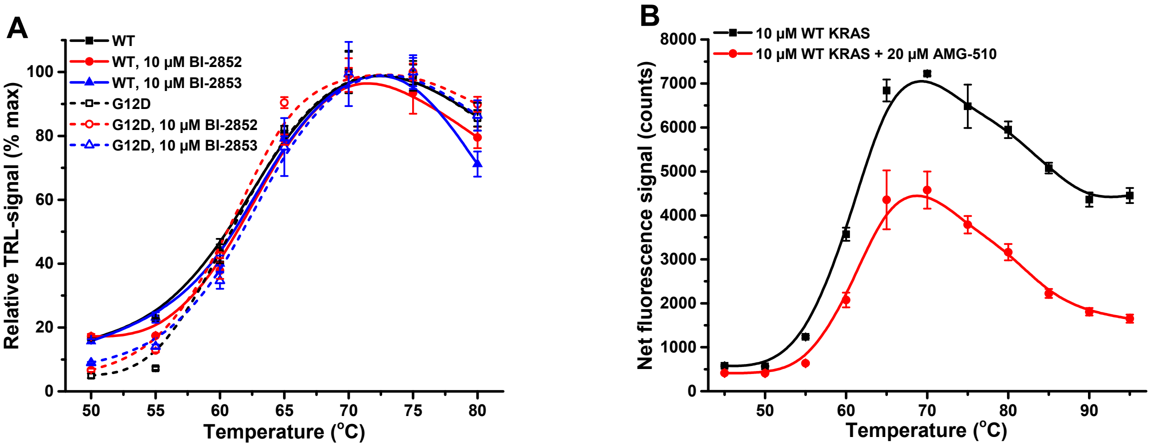


**Figure S8.** Thermal stability of KRAS WT and G12C with BI-2852 and BI-2853 and KRAS WT with AMG510 in the Protein-Probe and SYPRO Orange assays. **A**) In a Protein-Probe assay, the non-covalent inhibitor BI-2852 (red) and its non-binding control BI-2853 (blue) were assayed in 10 μM concentration with 50 nM KRAS WT (solid) or G12D (dashed). Neither compound altered the thermal stability of KRAS (black) when compared to the absence of compound. **B**) Covalent KRAS G12C targeting inhibitor AMG-510 showed quenching on the SYPRO Orange signal when assayed in 20 μM concentration with 10 μM KRAS WT (red) and compared to KRAS WT alone (black), but it did not alter the thermal stability of the KRAS WT. Data represent mean ± SD (n=3).

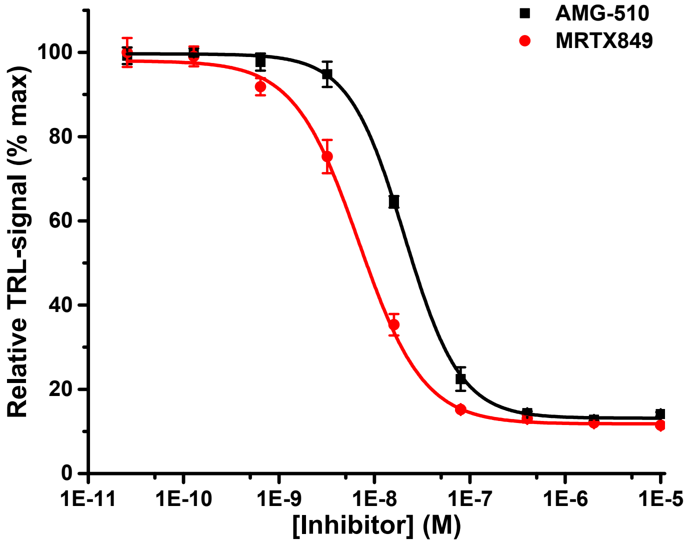


**Figure S9.** Nucleotide exchange inhibition of KRAS G12C with AMG-510 and MRTX849. Nucleotide exchange inhibition of KRAS G12C (15 nM) was tested in the presence of SOS$^{cat}$ (5 nM) by performing AMG-510 (black) and MRTX849 (red) titrations (0-10 µM). Both AMG-510 and MRTX849 showed a very potent binding giving $IC_{50}$ values of 20.7 ± 1.2 and 6.9 ± 0.8 nM, respectively. The final $IC_{50}$ values were reached after 10 min incubation, thus longer incubation provided no significant improvement. Data represent mean ± SD (n=3).

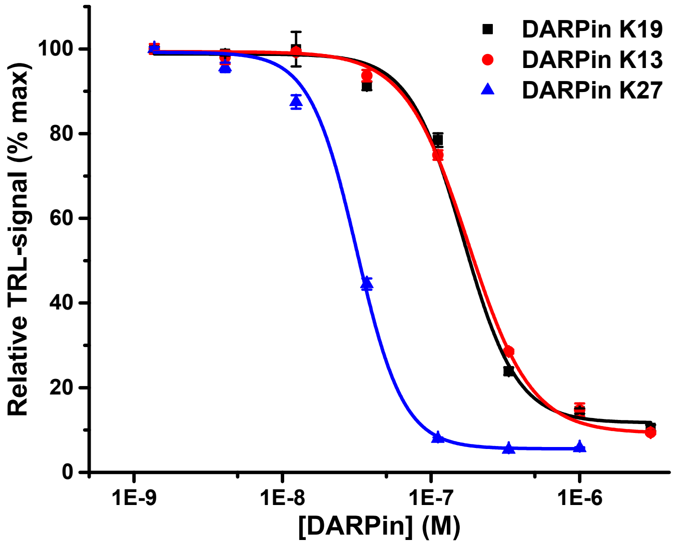


**Figure S10.** Nucleotide exchange inhibition in a SOS$^{cat}$ induced QRET activity assay with KRAS WT. Nucleotide exchange assay with KRAS WT (25 nM) was performed in the presence of 10 nM $Eu^{3+}$-GTP and monitored multiple times after SOS$^{cat}$ (10 nM) addition. Control DARPin (K27, blue) showed $IC_{50}$ value closely matching the one of KRAS concentration (25 nM), 30.6 ± 4.2 nM. $IC_{50}$ value for K19 and K13 were 125 ± 16 and 140 ± 23 nM, respectively. Data represents average ± SD (n=3).

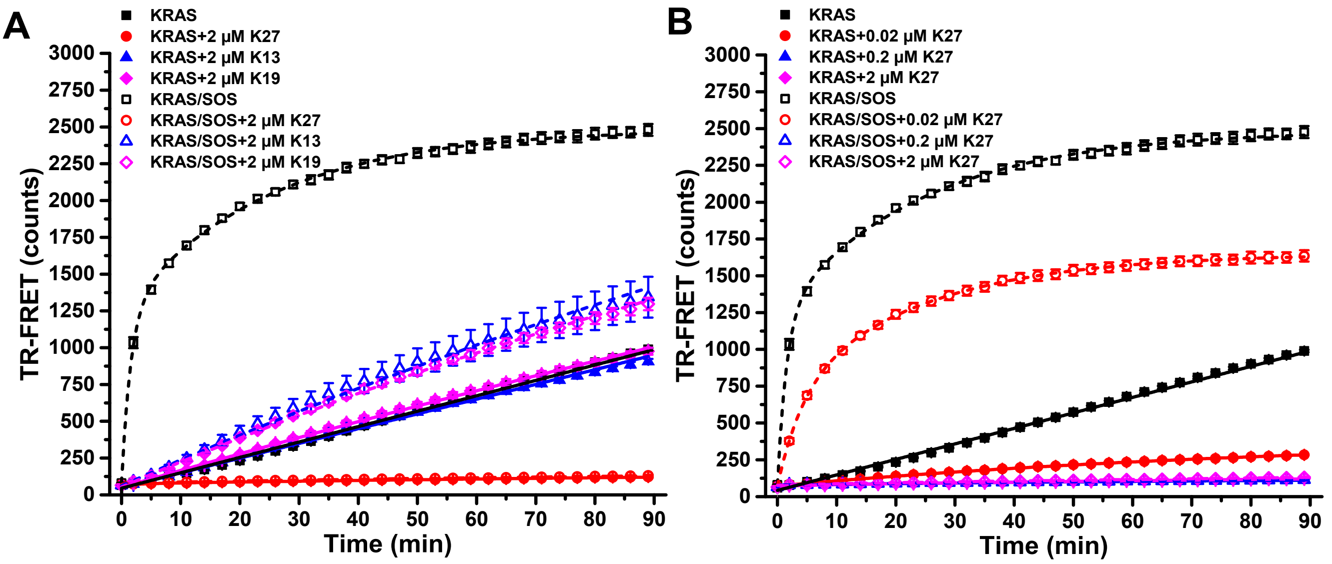


**Figure S11.** Inhibition of intrinsic and $SOS^{cat}$ induced nucleotide exchange monitored with Avi-KRAS. Nucleotide exchange assay with Avi-KRAS (50 nM) was performed in the presence of 50 nM AF647-GTP and monitored every 3 min after $SOS^{cat}$ and detection component addition (5 nM SA-$Eu^{3+}$). **A**) DARPins K13 (2 μM, blue), K19 (2 μM, magenta), and K27 (2 μM, red) were kinetically assayed with (open symbol) or without (solid symbol) $SOS^{cat}$ (5 nM) for 90 min, using KRAS without DARPin (black) as a control. DARPin K27 showed complete blocking of both intrinsic and $SOS^{cat}$ induced nucleotide exchange, as K19 and K13 only blocked $SOS^{cat}$ induced exchange and had no effect on intrinsic exchange. **B**) DARPin K27 was studied in three concentrations, 0.02 (red), 0.2 (blue), and 2 μM (magenta) with (open symbol) or without (solid symbol) $SOS^{cat}$ (5 nM), and using KRAS without DARPin (black) as a control. K27 completely blocks both $SOS^{cat}$ induced and intrinsic nucleotide exchange at 0.2 μM concentration. Data represents average ± SD (n=3).

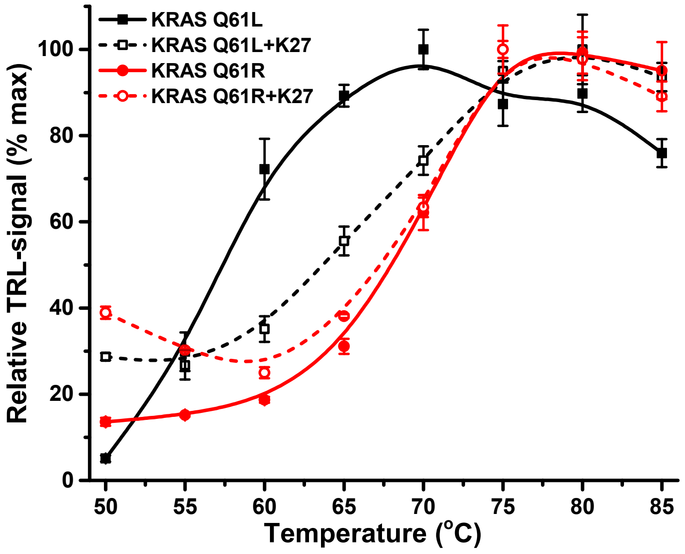


**Figure S12.** Thermal stability of KRAS Q61L and Q61R in the presence or absence of K27. DARPin K27 (100 nM) showed clear thermal stability increase with 50 nM KRAS Q61L (black) but not with Q61R in the Protein-Probe measurements. The increase in thermal stability produced by K27 with Q61L was $\Delta T_m$ = 9.4 °C, as in case of Q61R the stability increase with K27 was negligible ($\Delta T_m$ = 0.3 °C). Data represent mean ± SD (n=3).

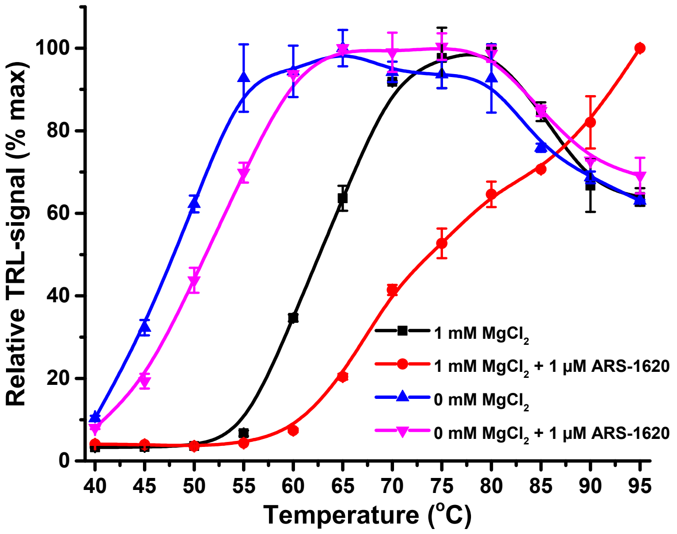


**Figure S13.** Buffer-induced effect on KRAS stability increase in the presence of ARS-1620. The ARS-1620 (1 µM) induced thermal stabilization of KRAS G12C (50 nM) is $Mg^{2+}$ dependent. In a buffer with 1 mM $MgCl_2$, ARS-1620 thermally stabilizes KRAS (red) over KRAS in its absence (black). Lack of $Mg^{2+}$ diminished the thermal stability effect, comparing KRAS alone (blue) with the KRAS:ARS-1620 complex (magenta). This demonstrates that ARS-1620 cannot bind or stabilize apo-KRAS formed in the absence of $MgCl_2$. Data represent mean ± SD (n=3).

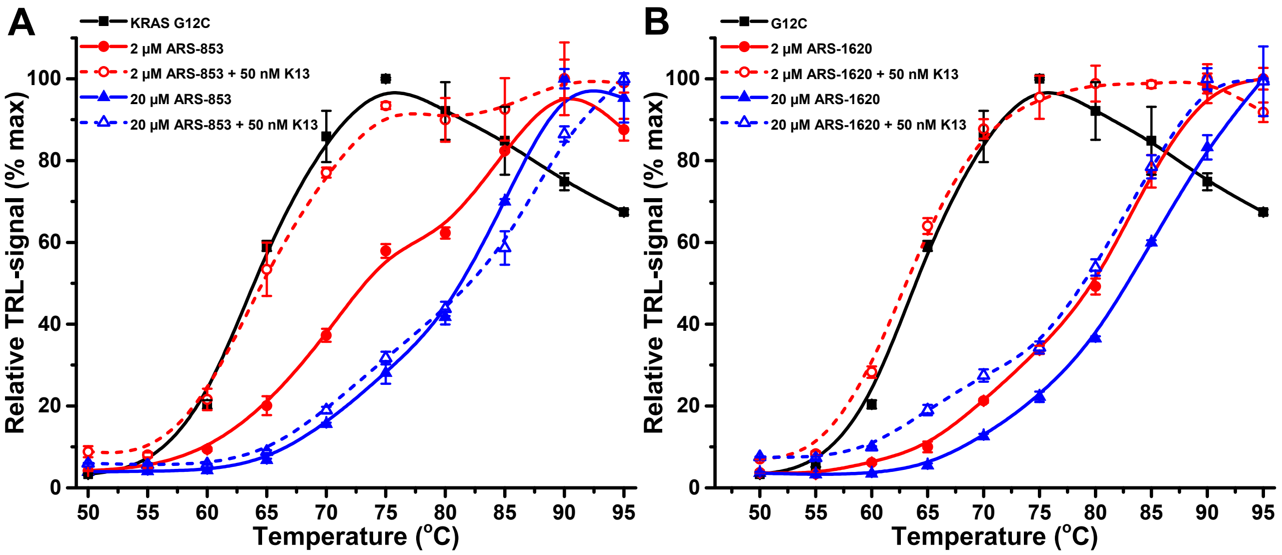


**Figure S14.** Competitive thermal shift assay for visualizing inhibitors having no effect on KRAS thermal stability. In the absence of K13 (solid), 50 nM KRAS G12C (black) was significantly stabilized by 2 (red) or 20 µM (blue) ARS-853 (**A**) and ARS-1620 (**B**). In the presence of 50 nM K13 (dashed), the stabilizing effect of both ARS-853 and ARS-1620 could be totally or partially blocked. In the case of 2 µM ARS-853 and ARS-1620, no thermal stability increase was visualized, indicating complete blocking of covalent attachment by K13. Higher inhibitor concentration could outcompete K13, increasing the KRAS G12C thermal stability close to that observed as without K13. Data represent mean ± SD (n=3).

**Table S1.** Small GTPase thermal stability using comparable conditions in the Protein-Probe assay.

| Protein | Concentration | Thermal Stability (°C) | | | | | | | |
|---|---|---|---|---|---|---|---|---|---|
| | | EDTA | $MgCl_2$ | GDP | GTP | GTPγS | GMP-PNP | GMP-PCP | GMP |
| | | 0.5 mM | 1 mM | 10 mM | 10 μM | 10 μM | 300 μM | 300 μM | 1 mM |
| **KRAS WT** | **50 nM** | 49.0 ± 0.3 | 61.3 ± 0.5 | 68.0 ± 0.5 | 68.2 ± 0.5 | 67.8 ± 0.6 | 67.1 ± 0.1 | 64.4 ± 0.4 | 61.3 ± 0.6 |
| **KRAS G12C** | **50 nM** | 48.2 ± 0.4 | 61.8 ± 0.5 | n.d.[a] | n.d. | n.d. | n.d. | n.d. | n.d. |
| **KRAS G12D** | **50 nM** | n.d. | 60.6 ± 0.5 | 67.6 ± 0.4 | n.d. | n.d. | n.d. | n.d. | n.d. |
| **KRAS G13D** | **50 nM** | 47.7 ± 0.5 | 50.7 ± 0.2 | 60.5 ± 0.2 | 60.8 ± 0.4 | n.d. | n.d. | n.d. | n.d. |
| **KRAS Q61R** | **50 nM** | 51.8 ± 0.9 | 71.9 ± 0.6 | 71.3 ± 0.9 | 71.7 ± 0.5 | n.d. | n.d. | n.d. | n.d. |
| **KRAS Q61L** | **50 nM** | n.d. | 60.4 ± 0.5 | n.d. | n.d. | n.d. | n.d. | n.d. | n.d. |
| **Protein, concentration** | | **EDTA** | **$MgCl_2$** | **GDP** | **GTP** | **GTPγS** | **GMP-PNP** | **GMP-PCP** | **GMP** |
| **KRAS WT** | **1 μM** | n.d. | 54.4 ± 0.6 | n.d. | n.d. | n.d. | n.d. | n.d. | n.d. |
| **KRAS V14I** | **1 μM** | n.d. | 47.5 ± 0.2 | n.d. | n.d. | n.d. | n.d. | n.d. | n.d. |
| **iMet-KRAS** | **1 μM** | n.d. | 66.9 ± 0.2 | n.d. | n.d. | n.d. | n.d. | n.d. | n.d. |
| **Ac-KRAS** | **1 μM** | n.d. | 66.0 ± 1.0 | n.d. | n.d. | n.d. | n.d. | n.d. | n.d. |
| **HRAS** | **1 μM** | n.d. | 64.4 ± 0.2 | n.d. | n.d. | n.d. | n.d. | n.d. | n.d. |
| **NRAS** | **1 μM** | n.d. | 59.4 ± 0.2 | n.d. | n.d. | n.d. | n.d. | n.d. | n.d. |
| **RhoA** | **1 μM** | 44.7 ± 0.4 | 61.3 ± 0.3 | 70.1 ± 1.1 | 73.0 ± 1.5 | n.d. | n.d. | n.d. | n.d. |

[a] Not determined (n.d.) in comparable conditions.

**Table S2.** KRAS nucleotide exchange activity inhibition using a panel of nucleotides and inhibitors.

| Inhibitor/Effector | SOS[cat] | KRAS WT ($EC_{50}/IC_{50}$) | | | KRAS G12C ($EC_{50}/IC_{50}$) | |
|---|---|---|---|---|---|---|
| | | 15 nM[a] | 25 nM | 100 nM | 15 nM | 100 nM |
| **GTP** | **5 nM** | 7.9 ± 0.6 nM[b] | n.d.[c] | n.d. | n.d. | n.d. |
| **GDP** | **5 nM** | 11.9 ± 0.7 nM[b] | n.d. | n.d. | n.d. | n.d. |
| **GTPγS** | **5 nM** | 11.7 ± 0.8 nM[b] | n.d. | n.d. | n.d. | n.d. |
| **GMP-PNP** | **5 nM** | 71.2 ± 9.3 nM | n.d. | n.d. | n.d. | n.d. |
| **GMP-PCP** | **5 nM** | 542 ± 38 nM | n.d. | n.d. | n.d. | n.d. |
| **GMP** | **5 nM** | 101 ± 8 μM | n.d. | n.d. | n.d. | n.d. |
| **AMG-510** | **5 nM** | n.d. | n.d. | n.d. | 20.7 ± 1.2 nM[b] | n.d. |
| **MRTX849** | **5 nM** | n.d. | n.d. | n.d. | 6.9 ± 0.8 nM[b] | n.d. |
| **BAY-293[d]** | **10 nM[e]** | n.d. | n.d. | 11.5 ± 2.2 nM | n.d. | 17.5 ± 3.0 nM |

| | | | | | | |
|---|---|---|---|---|---|---|
| **BI-2852** | **10 nM** | n.d. | n.d. | 2.8 ± 0.7 μM | n.d. | n.d. |
| **ARS-853** | **10 nM** | n.d. | n.d. | n.d. | n.d. | 1.1 ± 0.1 μM |
| **ARS-1620** | **10 nM** | n.d. | n.d. | n.d. | n.d. | 0.21 ± 0.04 μM |
| **DARPin K27** | **10 nM** | n.d. | 30.6 ± 4.2 nM | n.d. | n.d. | n.d. |
| **DARPin K13** | **10 nM** | n.d. | 140 ± 23 nM | n.d. | n.d. | n.d. |
| **DARPin K19** | **10 nM** | n.d. | 125 ± 16 nM | n.d. | n.d. | n.d. |

[a] KRAS concentration varied in different assays; [b] Values close to KRAS concentration do not reflect through $EC_{50}$/$IC_{50}$ value; [c] Not determined (n.d.) in comparable conditions; [d] BAY-293 is SOS inhibitor and do not show KRAS mutation specific action; [e] SOS concentration has no effect on $EC_{50}$/$IC_{50}$ values determined using KRAS binders.

## Supplemental References

1. Kopra, K.; Ligabue, A.; Wang, Q.; Syrjänpää, M.; Blaževitš, O.; Veltel, S.; van Adrichem, A.J.; Hänninen, P.; Abankwa, D.; Härmä, H. A homogeneous quenching resonance energy transfer assay for the kinetic analysis of the GTPase nucleotide exchange reaction. *Anal. Bioanal. Chem.* **2014**, 406, 4147-4156. doi: 10.1007/s00216-014-7795-7.
2. Kopra, K.; Vuorinen, E.; Abreu-Blanco, M.; Wang, Q.; Eskonen, V.; Gillette, W.; Pulliainen, A.T.; Holderfield, M.; Härmä, H. Homogeneous dual-parametric-coupled assay for simultaneous nucleotide exchange and KRAS/RAF-RBD interaction monitoring. *Anal. Chem.* **2020**, 92, 4971-4979. doi: 10.1021/acs.analchem.9b05126.
3. Syrjänpää, M.; Vuorinen, E.; Kulmala, S.; Wang, Q.; Härmä, H.; Kopra, K. QTR-FRET: Efficient background reduction technology in time-resolved förster resonance energy transfer assays. *Anal. Chim. Acta.* **2019**, 27, 1092-10101. doi: 10.1016/j.aca.2019.09.045.
4. Vuorinen, E.; Valtonen, S.; Eskonen, V.; Kariniemi, T.; Jakovleva, J.; Kopra, K.; Härmä, H. Sensitive label-free thermal stability assay for protein denaturation and protein-ligand interaction studies. *Anal. Chem.* **2020**, 92, 3512-3516. doi: 10.1021/acs.analchem.9b05712.
5. Ashok, Y.; Miettinen, M.; de Oliveira, D.K.H.; Tamirat, M. Z.; Näreoja, K.; Tiwari, A.; Hottiger, M.O.; Johnson, M.S.; Lehtiö, L.; Pulliainen, A.T. Discovery of compounds inhibiting the ADP-ribosyltransferase activity of pertussis toxin. *ACS Infect. Dis.* **2019**, 6, 588-602. doi: 10.1021/acsinfecdis.9b00412.
6. Bera, A.K.; Lu, J.; Wales, T.E.; Gondi, S.; Gurbani, D.; Nelson, A.; Engen, J.R.; Westover, K.D. Structural basis of the atypical activation mechanism of $KRAS^{V14I}$. *J. Biol. Chem.* **2019**, 294, 13964-13972. doi: 10.1074/jbc.RA119.009131.
7. Bery, N.; Legg, S.; Debreczeni, J.; Breed, J.; Embrey, K.; Stubbs, C.; Kolasinska-Zwierz, P.; Barrett, N.; Marwood, R.; Watson, J.; Tart, J.; Overman, R.; Miller, A.; Phillips, C.; Minter, R.; Rabbitts, T. KRAS-specific inhibition using a DARPin binding to a site in the allosteric lobe. *Nat. Commun.* **2019**, 10, 2607. doi: 10.1038/s41467-019-10419-2.
8. Dharmaiah, S.; Tran, T.H.; Messing, S.; Agamasu, C.; Gillette, W.K.; Yan, W.; Waybright, T.; Alexander, P.; Esposito, D.; Nissley, D.V.; McCormick, F.; Stephen, A.G.; Simanshu, D.K. Structures of N-terminally processed KRAS provide insight into the role of N-acetylation. *Sci. Rep.* **2019**, 9, 10512. doi: 10.1038/s41598-019-46846-w.
9. Taylor, T.; Denson, J-P.; Esposito, D. Optimizing expression and solubility of proteins in E. coli using modified media and induction parameters. *Methods Mol. Biol.* **2017**, 1586, 65-82. doi: 10.1007/978-1-4939-6887-9_5.
10. Kopra, K.; van Adrichem, A.J.; Salo-Ahen, O.M.H.; Peltonen, J.; Wennerberg, K.; Härmä, H. High-Throughput dual screening method for Ras activities and inhibitors. *Anal. Chem.* **2017**, 89, 4508-4516. doi: 10.1021/acs.analchem.6b04904.
11. Kopra, K.; Härmä, H. Methods to monitor K-Ras activation state. In Prior, I.; Rubio, I. (Eds.), Ras activity and signaling: methods and protocols. *Methods Mol. Biol.*, Springer US. **2021**, 137-167. doi: 10.1007/978-1-0716-1190-6_8.
12. Hunter, J.C.; Gurbani, D.; Ficarro, S.B.; Carrasco, M.A.; Lim, S.M.; Choi, H.G.; Xie, T.; Marto, J.A.; Chen, Z.; Gray, N.S.; Westover, K.D. In situ selectivity profiling and crystal structure of SML-8-73-1, an active site inhibitor of oncogenic K-Ras G12C. *Proc. Natl Acad. Sci. USA.* **2014**, 111, 8895-8900. doi: 10.1073/pnas.140463911.
